# Structure-preserving visualisation of high dimensional single-cell datasets

**DOI:** 10.1038/s41598-019-45301-0

**Published:** 2019-06-20

**Authors:** Benjamin Szubert, Jennifer E. Cole, Claudia Monaco, Ignat Drozdov

**Affiliations:** 1 Bering Limited, London, United Kingdom; 2 Kennedy Institute of Rheumatology, Nuffield Department of Orthopaedics, Rheumatology and Musculoskeletal Sciences, University of Oxford, Oxford, OX3 7FY UK

**Keywords:** Genomics, Computational biology and bioinformatics

## Abstract

Single-cell technologies offer an unprecedented opportunity to effectively characterize cellular heterogeneity in health and disease. Nevertheless, visualisation and interpretation of these multi-dimensional datasets remains a challenge. We present a novel framework, ivis, for dimensionality reduction of single-cell expression data. ivis utilizes a siamese neural network architecture that is trained using a novel triplet loss function. Results on simulated and real datasets demonstrate that ivis preserves global data structures in a low-dimensional space, adds new data points to existing embeddings using a parametric mapping function, and scales linearly to hundreds of thousands of cells. ivis is made publicly available through Python and R interfaces on https://github.com/beringresearch/ivis.

## Introduction

Characterising cellular composition is crucial for defining functional heterogeneity in health and disease^1^. The advent of technologies that interrogate genome-scale molecular information at single-cell resolution provides an unprecedented opportunity for systematic investigation at the level of DNA^2,3^, RNA^4^, proteins^5^, and metabolites^6^. Indeed, increasing utilization of these technologies has facilitated characterisation of previously unknown cell types^7,8^ developmental lineages^9^ and patterns of cellular organization^10^.

Visualisation and interpretation of single-cell experiments are underpinned by dimensionality reduction (DR) techniques. Non-linear approaches, including the t-distributed Stochastic Neighbor Embedding (t-SNE) algorithm^11^, have been shown to effectively capture complex data structures, outperforming linear projection methods such as Principal Component Analysis (PCA)^12,13^ Nevertheless, t-SNE has several limitations^14,15^. First, t-SNE is not robust in the presence of technical noise and tends to form spurious clusters from randomly distributed data points^14^, producing misleading results that may hinder biological interpretation. Second, due to non-parametric nature of t-SNE, addition of new data points to existing embeddings is not possible^11,15^. Instead, t-SNE needs to be rerun on the combined dataset, which is computationally expensive and not scalable. Third, t-SNE has a time complexity of *O*(*N*^2^*D*) and space complexity of *O*(*N*^2^), where *N* is the number of observations and *D* is the number of features in the data^11,15,16^. Whilst complexity can be reduced to *O*(*N log N*) by approximating the gradient using tree-based algorithms^17^, dimensionality reduction across tens of thousands of exemplars remains challenging. Finally, t-SNE preserves the local clustering structures^15^, but global structures such as inter-cluster relationships and distances cannot be reliably preserved^18^. As such, the biological information that may be extracted through t-SNE embeddings remains limited.

Neural Network (NN) models have been proposed as effective non-linear DR techniques^15,19,20^. Generally, unsupervised NNs with multiple layers are trained by optimizing a target function, whilst an intermediate layer with small cardinality serves as a low dimensional representation of the input data^19,21^. In this paper we introduce a scalable algorithm, ivis, which effectively captures local as well as global features of high-dimensional datasets. Additionally, ivis learns a parametric mapping from the high-dimensional space to low-dimensional embedding, facilitating seamless addition of new data points to the mapping function. Importantly, we demonstrate that ivis preserves distances in low-dimensional projections, enabling biological interpretation. We validate our method using synthetic, cytometry by time of flight (CyTOF), and scRNA-seq datasets.

## Results

### ivis benchmarks on synthetic datasets

**Figure 1:**
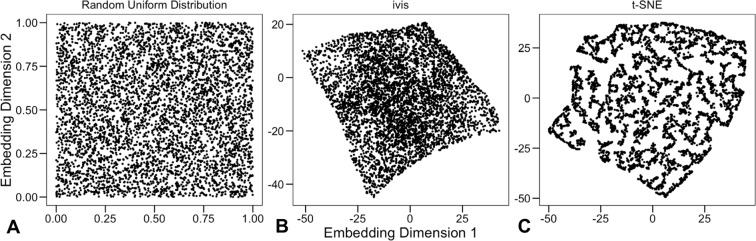
Benchmarking ivis and t-SNE on 5,000 uniformly distributed random data-points. (**A**) Original two-dimensional data. (**B**) ivis embedding of the nine-dimensional dataset. (**C**) t-SNE embedding of the nine-dimensional dataset.

**Figure 2:**
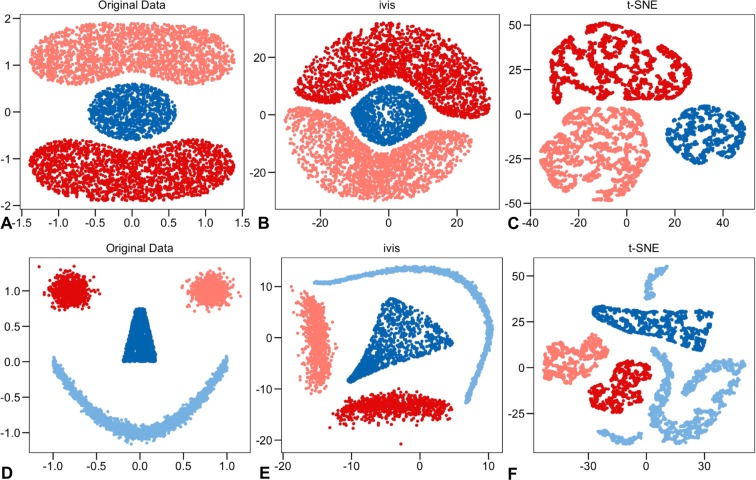
Benchmarking ivis and t-SNE on two synthetic datasets. (**A**,**D**) The original two-dimensional dataset consisting of 5,000 points, colored by cluster labels. (**B**,**E**) ivis embedding of the nine-dimensional dataset. (**C**,**F**) t-SNE embedding of the nine-dimensional dataset.

To demonstrate that ivis can uncover the global structure of a high-dimensional dataset, we first generated three synthetic datasets - random uniform noise, Cassini problem, and Smiley dataset (Figs 1A, 2A,D). The Cassini problem is a two-dimensional dataset with three clusters containing uniformly distributed data points. The smiley dataset consists of two Gaussian eyes, a trapezoid nose, and a parabola mouth with vertical Gaussian noise. Two-dimensional coordinates (x, y), were mapped to a nine-dimensional space by the transformation (x + y, x − y, xy, x^2^, y^2^, x^2^y, xy^2^, x^3^, y^3^)^15^. The nine-dimensional datasets were used as inputs to ivis and t-SNE algorithms.

Visual assessment suggests that ivis preserves random distributions of the original dataset (Fig. 1B). However, t-SNE groups random points into multiple compact clusters with clear boundaries (Fig. 1C). Subsequently, we assessed the capacity of ivis and t-SNE algorithms to extract inter-cluster relationships. Whilst both ivis and t-SNE uncovered the three clusters in the Cassini dataset (Fig. 2A–C), t-SNE did not preserve inter-cluster relationships. Additionally, increasing cluster complexity using the Smiley dataset (Fig. 2D–F), demonstrated that ivis preserves both the shape and relative locations of each cluster in the embedding space (Fig. 2E). In contrast, t-SNE embeddings yielded additional spurious clusters and complete loss of all inter-cluster relationships (Fig. 2F).

ivis utilizes several stochastic processes - namely approximate identification of the k -nearest neighbors (KNNs) using random projection trees and random initialisation of neural network weights. As such, the low-dimensional data representation may change across multiple ivis runs. To test stability of the two-dimensional embedding, we ran ivis ten times on the Smiley benchmark dataset (Supplementary Fig. S2). The two-dimensional structure across all ten runs was consistently preserved. Conversely, cluster layout and organization changed drastically for each t-SNE run (Supplementary Fig. S3).

### Single-cell CyTOF datasets

**Figure 3:**
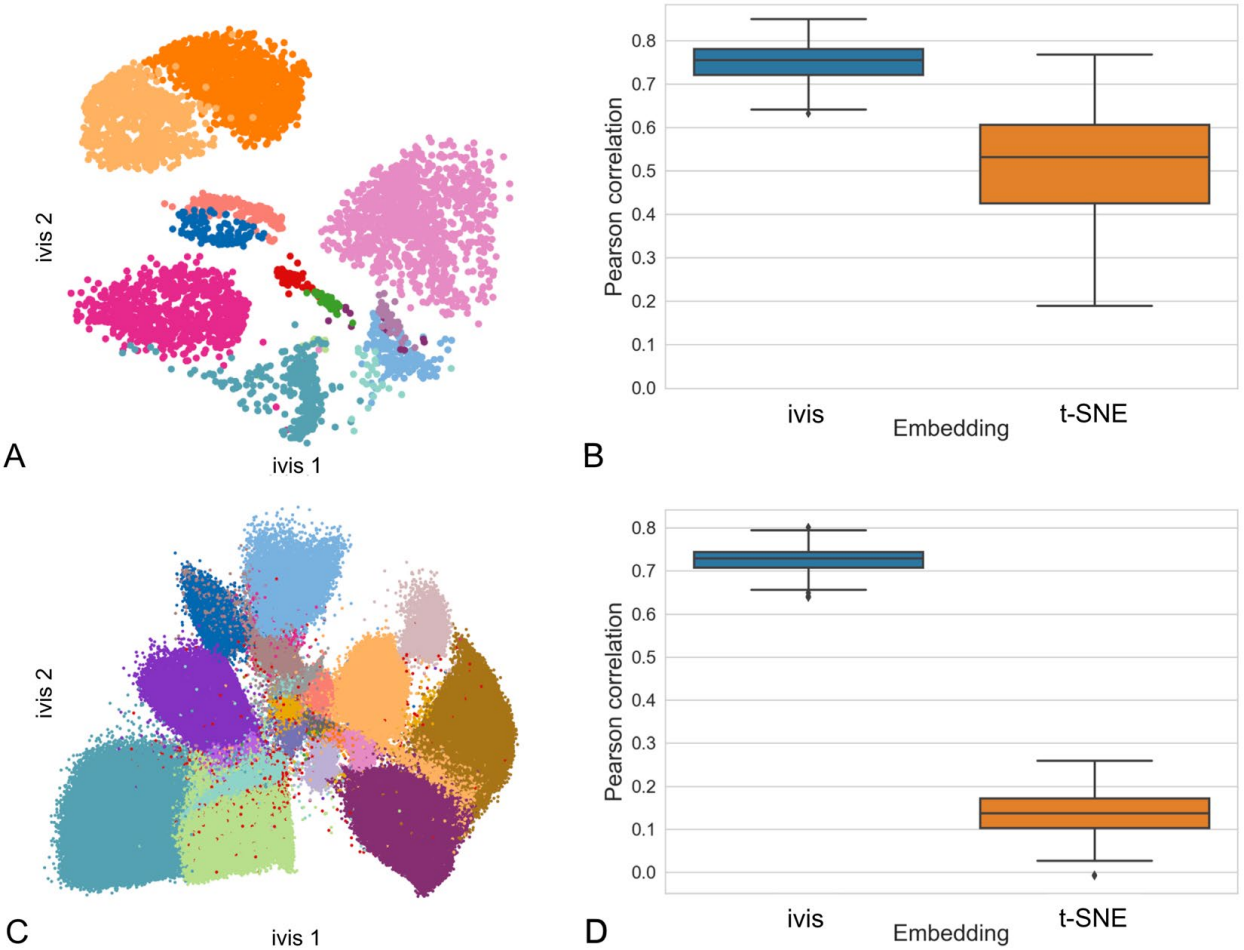
Phenotypic characterisation of healthy human BMMCs. (**A**) ivis display of 10,000 cells from healthy BMMC benchmark data. Cells are colored by cell-type assignments established by manual gating. (**B**) Boxplot of Pearson’s correlation coefficients (PCC) between centroids of manual gates in the full-dimensional data and centroids of those same points in either ivis or t-SNE embedding (median PCC_ivis_ = 0.76, median PCC_t-SNE_ = 0.53). (**C**) ivis display of all cells from the Samusik dataset. Cells are colored by cell-type assignments established by manual gating. (**D**) Boxplot of PCCs between centroids of manual gates in the full-dimensional data and centroids of those same points in either ivis or t-SNE embedding (median PCC_ivis_ = 0.73, median PCC_t-SNE_ = 0.13).

The capacity of ivis to uncover structure in single cell experiments was evaluated using two CyTOF datasets. First, the human BMMC and mouse bone marrow (Samusik) datasets were reduced to two ivis dimensions and cellular populations were identified using phenograph^22^ clustering of the two-dimensional embeddings. Phenograph identified 12 and 25, clusters in the BMMC and Samusik dataset respectively, which exhibited high concordance with manual gates (adjusted Rand Index_BMMC_ = 0.97, Fig. 3A, adjusted Rand Index_Samusik_ = 0.45, Fig. 3C). To establish how well ivis and t-SNE preserve global features, a Euclidean distance matrix between centroids of the manually-gated cells was created for the original data, the ivis embeddings, and the t-SNE embeddings. The level of correlation between the original distance matrix and the distance matrices in the embedding spaces was then assessed using the Mantel test (see Methods). This process was repeated for one hundred random subsamples of the data (n = 10,000 cells per subsample selected without replacement) to generate a distribution of correlation values. Cluster centroid distances in the ivis space were significantly correlated with the original dataset using the Pearson’s Correlation Coefficient (PCC) (median PCC_ivis-BMMC_ = 0.76 vs. median PCC_t-SNE-BMMC_ = 0.53, p-value ≪ 0.01, Fig. 3B, median PCC_ivis-Samusik_ = 0.73 vs. median PCC_t-SNE-Samusik_ = 0.14, p-value ≪ 0.01, Fig. 3D).

**Figure 4:**
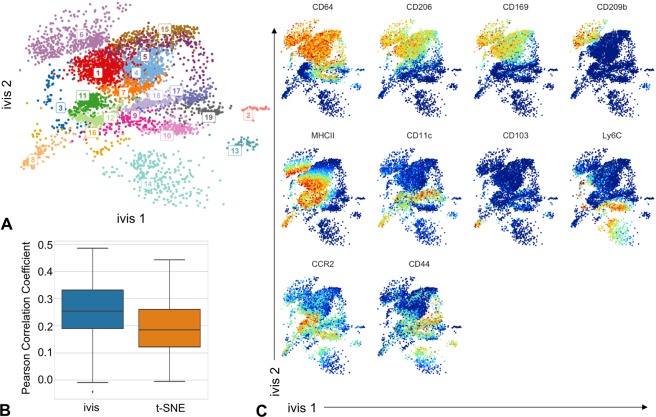
Visualisation of myeloid cells from ApoE^−/−^ mice. (**A**) Two-dimensional ivis embedding of a random sub-sample of 10,000 cells. Each cell is coloured according to a unique cluster identified by the application of phenograph algorithm to the full 21-dimensional dataset. (**B**) Boxplot of Pearson’s correlation coefficients between centroids of gates in the full-dimensional data and centroids of those same points in both ivis and t-SNE embeddings across one-hundred random subsamples of the data. Median PCC_ivis_ = 0.25 vs. median PCC_t-SNE_ = 0.18. (**C**) Heatmap overlay that displays how marker expression and intensity profiles express in monocyte and macrophage populations.

The healthy human BMMC and the Samusik datasets are well-characterised benchmarks for dimensionality reduction problems, mainly due to the highly informative features (cellular markers) within each dataset. In practice, feature selection is an integral part of the discovery process and often CyTOF datasets comprise both informative as well as noisy markers. To assess how well the ivis methodology performs on a typical discovery dataset, we applied the ivis algorithm to 21 markers in myeloid cells collected from aortas of Apoe^−/−^ mice (see Methods). Two-dimensional ivis embedding preserved phenograph-derived clusters of the full dataset (Fig. 4A), whilst better retaining the global inter-cluster distances as compared to t-SNE (median PCC_ivis_ = 0.25 vs. median PCC_t-SNE_ = 0.18, t-statistic = 4.50, p-value ≪ 0.01, Fig. 4B).

### Single-cell RNAseq datasets

Given the relatively low dimensionality of CyTOF datasets (typically tens of features), we investigated whether the ivis algorithm is also applicable to scRNA-seq experiments that contain thousands of features. Due to the high-throughput nature of these datasets, we used PCA as a noise-reducing pre-processing step^12^, projecting all cells to 50 Principal Components prior to embedding with ivis.

**Figure 5:**
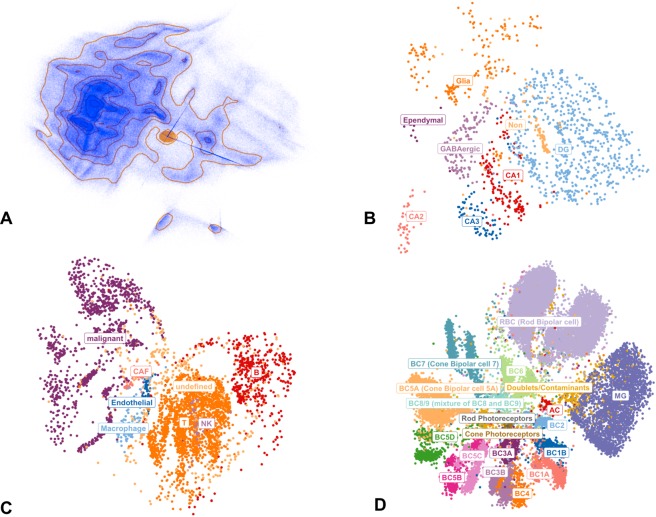
Structure-preserving dimensionality reduction of single cell transcriptomes using ivis. (**A**) The 10x genomics mouse brain dataset (n = 1.3 million cells). Contours represent dense regions in the embedding space. (**B**) The hippocampus dataset (n = 1,402 cells. (**C**) The melanoma dataset (n = 4,645 cells). (**D**) Mouse retinal bipolar neural cells (n = 27,499 cells) In all cases, each cell is colored by its cell type.

First, we assessed the scalability of ivis using 1.3 million cells from the 10X genomics mouse dataset^23^. The scikit-learn Barnes-Hut t-SNE implementation did not finish analysis within 24 hours and was terminated. Conversely, we were able to obtain meaningful ivis embeddings without subsampling in <30 minutes (Fig. 5A).

Projection of the hippocampus dataset into two-dimensional ivis space revealed distinct nuclei clusters that corresponded to known cell types and anatomical regions in the hippocampus (Fig. 5B). Importantly, ivis captured the flow of sensory information within the hippocampus from the dentate gyrus (DG) to CA3 and CA1 nuclei, as exemplified by the mutual cell proximities in these clusters. Additionally, functional dissimilarity between CA2 and CA3 was highlighted through more distal positioning of these nuclei in the embedding space.

Similarly, analysis of intra-tumor heterogeneity in metastatic melanoma revealed that normal and malignant cells formed distinct clusters (Fig. 5C). Interestingly, normal immune cells, such as T cells, B cells, and macrophages originating from different individuals, were grouped together by cell type rather than origin. Importantly, Cancer Associated Fibroblasts (CAF) were found to be adjacent to both normal and malignant cells.

Finally, ivis embeddings of the retinal bipolar dataset showed clear segregation between non-bipolar (amacrine cells [AC], photoreceptors Mueller glia [MG]) and bipolar (rod and cone bipolar cells) cells (Fig. 5D). Furthermore, the “off” cone bipolar cells (BC1A, BC1B, BC2, BC3A, BC3B, BC4) and the ‘on’ cone bipolar cells (BC5A-D, BC6, BC7, BC8/9) were localised to two distinct regions of the embedding space, exhibiting a direct correlation between biological function and embedding proximities. Finally, doublets and contaminants (2.4% of the dataset) were reliably grouped together, despite being a low-frequency population.

### Learning embeddings for single-cell datasets

**Figure 6:**
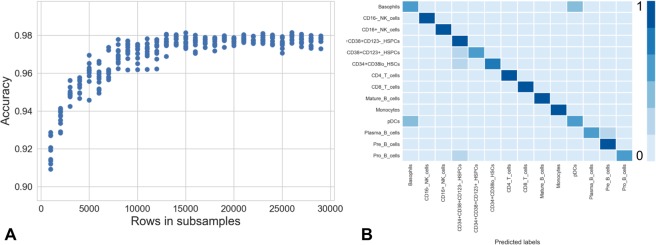
Random forest classifier performance on ivis embeddings inferred from independent subsets of healthy human BMMC data. (**A**) Scatterplot depicting accuracy of a random forest classifier when trained on embedded subsets of varying size. The experiments for each subset size were repeated ten times. (**B**) Confusion matrix for a single random forest classifier trained on a subset of 10,000 embedded data-points and validated on the remaining 94,184 points.

To assess whether our algorithm could be used to extrapolate embeddings to out-of-sample data points, the ivis model was trained on randomized subsets of the BMMC dataset (n = 1,000–30,000 rows in intervals of 1,000). This process was repeated ten times to generate a distribution for each subset size (Fig. 6A). Next, a random forest classifier was used to learn the mapping between two-dimensional embeddings and the corresponding manually defined cell populations. Finally, new two-dimensional embeddings were generated for the out-of-sample data points using ivis projections and predicted cell population labels were extracted using the pre-trained random forest. The accuracy of the random forest classifier increased with subsample size (Fig. 6A). Interestingly, the worst performing run (subsample size of 1,000) still achieved a classification accuracy of 0.91 on out-of-sample predictions, despite using less than 1% of the 104,184 data-points present in the dataset.

### Sensitivity to hyperparameters

ivis has several hyperparameters, such as margin (*m* in equation (2)), *k* (the number of nearest neighbors for positive and negative point selection), and loss function. We sought to evaluate whether ivis is resistant to variations in these values and subsequently identify sensible defaults for DR problems.

Systematically increasing *m* for three datasets (Cassini, Smiley, and BMMC, Supplementary Figs 4–6 in an interval (0, 1000] demonstrated that ivis embeddings with *m* in [0.1, 500] retained the shape of the original data. However, for *m* > 100, we noted increasing information loss in the embedding space, manifested through highly correlated ivis dimensions (Supplementary Fig. S4H–J, Supplementary Fig. S6G–J). Interestingly, for *m* > 500 we observed greater incidence of exploding gradients resulting in uninformative embeddings (Supplementary Fig. S5I).

To evaluate the effects of *k* on embedding accuracy, we subsampled the BMMC and Samusik datasets with subsample sizes in {1000, 2500, 5000, 10000, 20000, 50000} with *k* in {2, 4, 8, 16, 32, 64, 128, 256, 512, 1024, 2048, 4096}. For each combination of subsample and *k* we generated ivis embeddings which were used to train a random forest classifier that mapped embeddings to manual gates. Subsequently, for the remaining out-of-sample cells, we predicted ivis coordinates and corresponding cellular populations. Accuracy was assessed by comparing predicted population labels with manual gates. Although prediction accuracies were generally stable for 16 < k < 256 irrespective of subsample size, we observed that setting *k* to 0.5–1% of the number of observations consistently resulted in greater accuracies (Supplementary Fig. S7).

Finally, we assessed whether our variant of the triplet-loss function (pn loss, see Methods) presents an effective alternative to the conventional triplet loss and softmax-ratio loss functions^24^. For each subsample multiple loss functions were used to generate ivis embeddings, which were subsequently used to train a random forest classifier that mapped embeddings to manual gates. For the remaining out-of-sample cells (held out test set), we obtained predicted ivis embeddings and the corresponding cellular populations. Accuracy was assessed by comparing predicted population labels with manual gates. Overall, pn loss with a Euclidean distance metric outperformed other loss functions (Table 1). However, the Manhattan distance metric appeared to perform slightly better on the smallest subset (n = 1,000 data points).

**Table 1:** Out-of-sample accuracies of ivis embeddings generated using multiple loss and distance functions.

Loss	Subsample			
	n = 1,000	n = 5,000	n = 10,000	n = 15,000
Euclidean PN	0.94	**0**.**96**	**0**.**97**	**0**.**97**
Euclidean	0.93	0.95	0.95	0.95
Manhattan PN	**0**.**95**	0.96	0.96	0.96
Manhattan	0.93	0.95	0.96	0.96
Chebyshev PN	0.93	0.96	0.97	0.97
Chebyshev	0.92	0.95	0.96	0.96
Softmax Ratio PN	0.93	0.95	0.94	0.93
Softmax Ratio	0.9	0.92	0.96	0.93

## Discussion

In this work we present a novel algorithm for visualisation and interpretation of single-cell datasets. Our approach effectively captures higher orders of structure in a low-dimensional space by minimising a triplet-loss function (see Methods, Supplementary Fig. S1).

Our analysis using a synthetic dataset demonstrated that ivis is robust in the presence of uniform random noise. Given that high-throughput experiments are frequently subject to technical outliers^25–28^, we believe that ivis offers a realistic data representation framework. Although t-SNE is often a method of choice for visualization of single cell experiments^13,29^, we demonstrated that in the presence of random noise, the algorithm tends to yield spurious clusters with clean boundaries, potentially hindering accurate interpretation and discovery (Fig. 1C). This phenomenon has been recapitulated in other real-world, as well as synthetic, datasets^14,15^ and may be a general feature of algorithms that aim to preserve the pairwise (dis)similarities (e.g. LargeVis)^14^.

Using two synthetic and three CyTOF datasets, we have shown that ivis reduces dimensionality whilst preserving the “global” structure in a dataset. For example, in the synthetic Smiley dataset, ivis preserved both the shape and relative locations of each cluster in the embedding space. In contrast, t-SNE embedding resulted in emergence of spurious clusters and complete loss of overall inter-cluster relationships (Fig. 2F). Furthermore, in CyTOF experiments, ivis embeddings exhibited greater degrees of correlation with the original multi-dimensional data structures compared to t-SNE (Figs 3, 4). This is consistent with the capacity of t-SNE to effectively characterize the local neighborhood of each point in the original space and low-dimensional embedding at the expense of overall structure^30^. Given that ivis samples positive and negative data-points for each triplet, from the KNN vector and outside the KNN vector respectively, the choice of triplets at the time of training captures both local and global information of the data (see Methods).

Furthermore, structure-preserving properties of the ivis algorithm can greatly enhance discovery in single-cell datasets. For instance, ivis embeddings of the hippocampus dataset captured distinct nuclei clusters that correspond to known cell types and anatomical regions. Importantly, embedding regions of DG, CA3, and CA1 nuclei correctly reflect the flow of sensory information in the hippocampus^31^, suggesting that ivis is able to capture phenotypical variation in the data. In the metastatic melanoma dataset, malignant cells localised to the same ivis region, forming cluster based on the patient origin, whilst healthy immune cells from different patients clustered together by cell types. Importantly, CAF cells were localised adjacent to the malignant cells, further highlighting the phenotype-preserving characteristic of the ivis algorithm.

Single-cells experiments are increasingly used to define molecular characteristics and clinical outcomes in conditions such as cancer^32–34^ and atherosclerosis^35^. As these technologies become further integrated with precision medicine approaches, parametric methods that learn to generalize embeddings, without the need to be retrained, will become essential for scalable prediction of complex outcomes including response to treatment and patient survival^36^. We demonstrated that ivis requires as little as 1,000 cells (1% of the full dataset) to reliably (>90% accuracy) embed an out-of-sample dataset with 100,000 cells. Although conventional deep neural network approaches may require tens of thousands of exemplars to learn a generalizable set of parameters^37,38^, ivis employs a siamese neural network architecture^39^ that learns to discriminate between similar and dissimilar points without imposing strong priors. A variation of our approach has been previously applied to solve the one-shot learning problem for image recognition in which a network must correctly make predictions given only a single example of each new class^40^.

Whilst t-SNE remains a popular DR and visualization method, several algorithms have been introduced to improve either its computational performance or interpretability. The SIMLR algorithm improves upon t-SNE by learning a similarity matrix between cells, which is then used as an input to t-SNE for dimensionality reduction^41^. However, this approach is computationally expensive as the objective function involves an expensive multiplication of an N × N kernel matrix and N × N similarity matrix, where N is the number of cells^15^. Parametric t-SNE^11^ learns a parametric mapping from the high-dimensional space to a lower dimensional embedding. The method is generalizable to out-of-sample data and computes a loss function that minimizes Kullback-Leibler (KL) divergence between the point distributions in the original and the low-dimensional space. However, this approach does not preserve global distances and only local structures are captured by taking advantage of KL-divergence’s asymmetric properties^42^.

More recently, the scvis algorithm was introduced to facilitate interpretable dimensionality reduction for single-cell experiments^15^. The algorithm utilizes a Variational Autoencoder (VAE) with an additional regularization term that encourages the formation of gaps between clusters of data points. scvis was shown to preserve global structure of the high-dimensional measurements. The algorithm relies on obtaining the pairwise distances between two cells in a mini-batch during the training process, which takes *O*(*TN*^2^*D* + *TN*^2^*d*) time, where *N* is the mini-batch size, *D* is the dimensionality of the input data, *d* is the dimensionality of the low-dimensional latent variables, and *T* is the number of iterations. Conversely, ivis exhibits a linear time complexity *O*(*N*), where N is the dimensionality of the input data, due to selection of triplets without the need to pre-compute pairwise-distances (Supplementary Fig. S9).

Finally, the DeepCyTOF framework^43^ contains a denoising autoencoder component designed to handle missing data in CyTOF experiments. However, the framework facilitates semi-automatic gating and does not focus on data visualization.

In conclusion, we have developed a robust dimensionality reduction framework that retains global and local features of single-cell experiments in a low-dimensional space and is robust to hyperparameter settings. We demonstrate that ivis scales seamlessly to hundreds of thousands of cells, facilitating visualization and biological interpretation of complex features. As single-cell technologies continue to proliferate, we anticipate that ivis will offer a powerful computational approach for data visualization and discovery.

## Methods

### Neural network architecture and training

Structure-preserving dimensionality reduction is achieved using siamese neural networks (SNNs)^40^. SNNs are a class of neural network that employ a unique architecture to naturally rank similarity between inputs. The ivis SNN consists of three identical base networks (Supplementary Fig. S1A); each base network has three dense layers of 128 neurons followed by a final embedding layer. The size of the embedding layer reflects the desired dimensionality of outputs; results presented in this work utilize a final embedding layer with two neurons.

The layers preceding the embedding layer use the SELU activation function,

1 $$selu(x)=\lambda \{\begin{array}{lc}x & if\,x > 0\\ \alpha {e}^{x}-\alpha  & if\,x\le 0\end{array}$$

which gives the network a self-normalizing property^44^. The values for α and λ were set to 1.6733 and 1.0507 respectively^44^. The weights for these layers are randomly initialized with the LeCun normal distribution. The embedding layers use a linear activation and have their weights initialized using Glorot’s uniform distribution.

To regularize the network and prevent over-fitting, each dense layer is interleaved by Alpha Dropout layers with a dropout rate of 0.1; these layers randomly set a fraction of input units to 0 at each update, but are designed to work with SELU to maintain the property of self-normalization by maintaining the mean and variance of inputs.

The loss function used to train the network is a variant of the standard triplet loss function^41,45^:

2 $${L}_{tri}(\theta )={[\sum _{a,p,n}{D}_{a,p}-min({D}_{a,n},{D}_{p,n})+m]}_{+}$$

where *a*, *p*, and *n* correspond to anchor, positive, and negative points respectively, *D* is the Euclidean distance, and *m* is the margin. The Euclidean distance *D* (3) reflects similarity between points *a* and *b* in the embedding space.

3 $${D}_{a,b}=\sqrt{\sum _{i=1}^{n}{({a}_{i}-{b}_{i})}^{2}}$$

Although other distance metrics can be used, the Euclidean distance consistently outperforms other approaches and may be more interpretable from a biological standpoint (Table 1). Our implementation of the triplet loss function (pn loss) trains the network to satisfy the constraints of each triplet by simultaneously minimizing the Euclidean distance between the *anchor* (a point of interest) and the *positive* exemplar (a point similar to the Anchor) while maximizing the distance between the *anchor* and the *negative* exemplar (a point that is different from the anchor) (Supplementary Fig. S1B). This triplet constraint is said to be satisfied if the anchor point is closer to the positive point than to the negative point by a margin *m*. The pn loss function also takes into account the distance between the positive and the negative point by requiring the anchor and positive to be closer than the minimum between the anchor positive distance and the positive negative distance. This leads to a more robust loss function that improves separability in the embedding space (Supplementary Fig. S8) and avoids calculation of pairwise distances across a batch.

The triplet sampling procedure is as follows. Each triplet sampled from the dataset is made up of an anchor, a positive point that is similar to the anchor, and a negative point that is dissimilar to the anchor. The *k-*nearest neighbors (KNNs) are retrieved for each point in the dataset and a neighbor is randomly selected to be the positive example in the triplet. A random data-point outside of the *k-*nearest neighbors is used as the negative example. Setting *k* to an integer value between 0.5% and 1% of the number of observations appears to produce the most accurate embeddings (Supplementary Fig. S7) The triplets are generated dynamically during training, ensuring that each epoch contains different sets of triplets that reflect both local and global information of the data. The KNNs are estimated for each point using random projection trees implemented in the Annoy system^46^.

The SNN was trained on mini-batches of size 128 for 1000 epochs using the Adam optimizer function with a learning rate of 0.001 and standard parameters (${\beta }_{1}=0.9,{B}_{2}=0.999$). Training was halted early if the loss failed to decrease over 50 consecutive epochs.

### Performance assessment

To quantitate the degree to which ivis and t-SNE embeddings preserve the global structure of the data, we first cluster the original data, obtaining cluster centroids (average cluster expression vectors) and compute the inter-centroid distance matrix. Clusters are obtained either by using manual gating information or by applying the phenograph algorithm^22^ in cases where gold-standard cluster assignments are not provided. In all cases, phenograph clustering was applied using default hyperparameters. Next, we embed high-dimensional datasets into two-dimensional space using either ivis or t-SNE and calculate the distance matrix between cluster centroids within these embeddings. We then measure the Pearson Correlation Coefficient (PCC), with respective p-values, between centroid distance matrices in the original and embedding spaces using the Mantel test. This process was repeated on one hundred random subsamples of the dataset to generate a distribution of correlation values for both the ivis and t-SNE embeddings. Subsampling was carried out without replacement. Means of each distribution were compared using a two-tailed Student’s *t*-test.

### Learning a mapping function

To investigate whether a subset of cells is sufficient to extrapolate ivis embeddings to an out-of-sample dataset, we generated ivis coordinates for multiple small subsample of the dataset. All subsampling was performed without replacement. A supervised random forest classifier was then trained on the subset embeddings and respective cluster assignments. Subsequently, the ivis model was used to predict embeddings on out-of-sample data and the random forest classifier was used to infer the class of these embeddings. Classifier performance metrics on all out-of-sample predictions were subsequently obtained.

### Computational Complexity Analysis

To test the scalability of ivis, synthetic datasets of increasing size were generated and the required processing time to generate the ivis embeddings was measured. The synthetic datasets were 32-dimensional, with the number of rows doubling each iteration. The scikit-learn implementation of the Barnes-Hut t-SNE algorithm was also used to embed the datasets. All experiments were run on a server equipped with 32GB RAM and an Intel Xeon E5-2630 v3 processer with a clock speed of 2.40 GHz, using 12 of the 16 available logical threads.

### Single cell datasets

#### CyTOF

Three datasets were used for CyTOF evaluation. First, a 32-dimensional dataset consisting of protein expression levels of healthy human bone marrow mononuclear cells (BMMCs) from two healthy individuals^22^. Second, a 21-dimensional dataset of myeloid cell events from aortas of apolipoprotein E-deficient (ApoE^−/−^) mice fed either a chow or a high fat diet^47^.

The Samusik dataset^48^ is a 39-dimensional data set, consisting of 10 replicate bone marrow samples from C57BL/6J mice (samples from 10 different mice). Manually gated cell population labels were available for 24 immune cell populations. Cells not assigned to any population by manual gating were excluded from analysis.

In all cases, the arcsinh transform (scale factor 5) was applied to the raw FCS files^49^.

#### scRNA-seq

Four scRNA-seq datasets were included in this study. All data was downloaded from the single-cell portal^50^. For all the scRNA-seq datasets, we used PCA (as a noise-reduction preprocessing step^12^) to project the cells into a 50-dimensional space and used the projected coordinates in the 50-dimensional space as inputs to ivis^15^.

The 10X Genomics neural cell dataset consists of 1,306,127 cells from cortex, hippocampus, and subventricular zones of two E18 C57BL/6 mice^23^. The cells were sequenced on 11 Illumina Hiseq. 4000 machines to produce 98 bp reads^22^.

The adult mouse hippocampus consists of 1,402 single nuclei from hippocampal anatomical sub-regions (DG, CA1, CA2, and CA3), including enrichment of genetically-tagged lowly abundant GABAergic neurons^51^. The dataset contains high-quality outputs across animal age groups (including 2 years old mice), detecting 5,100 expressed genes per nucleus on average.

The melanoma dataset monitors expression of 4,645 cells isolated from 19 metastatic melanoma patients^52^. The cDNAs from each cell were sequenced by an Illumina NextSeq. 500 instrument to 30 bp pair-end reads with a median of ~150,000 reads per cell. The expression of each gene (23,686 genes in total) is quantified by log2 (TPM/10 + 1)^15^.

The bipolar dataset consists of 27,499 mouse retinal bipolar neural cells from a transgenic mouse interrogated using low-coverage (median depth of 8,200 mapped reads per cell) sequencing^12^. The dataset comprises of 15 clusters. Fourteen of these were assigned to bipolar cells and one cluster comprised of Mueller glia cells. These 15 clusters account for 96% of all the 27,499 cells. Doublets and contaminants (669 cells) account for 2.4% of all cells^15^.

## Supplementary information

Supplementary Information

## Data Availability

The ivis Python and R packages are available from github (https://github.com/beringresearch/ivis).
