# Supplementary material for: Structure-preserving visualisation of high dimensional single-cell datasets
Source: Sci Rep. 2019 Jun 20;9:8914. doi: 10.1038/s41598-019-45301-0 (PMC6586841; doi:10.1038/s41598-019-45301-0)
Supplement: Supplementary file 1 — Supplementary Information [file 41598_2019_45301_MOESM1_ESM.pdf]

Supplementary Information: Structure-preserving visualisation of high dimensional single-cell datasets

Benjamin Szubert[1], Jennifer E Cole[2], Claudia Monaco[2], Ignat Drozdov*[1]

1. Bering Limited, London, United Kingdom
2. Kennedy Institute of Rheumatology, Nuffield Department of Orthopaedics, Rheumatology and Musculoskeletal Sciences, University of Oxford, Oxford OX3 7FY, UK

*Address correspondence to:

idrozdov@beringresearch.com

Supplementary Figures

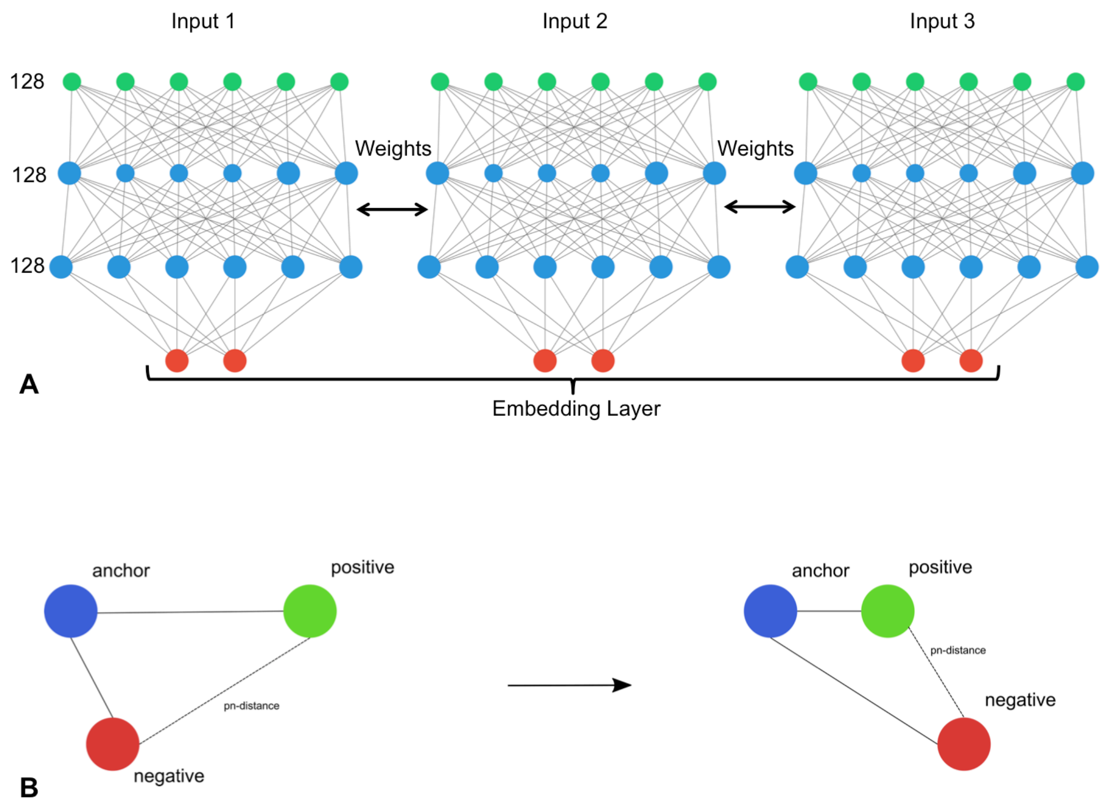

Supplementary Figure S1. Network architecture and training. A. Siamese network architecture consisting of three subnetworks with tied weights. Each subnetwork is made up of three densely connected layers with 128 neurons, followed by an embedding layer of two neurons. **B.** The pn-loss function was used to train the neural network. During the process of training, the anchor and positive point are pulled towards each other, while the anchor and negative and the positive and negative point are pushed away from each other. The objective of a pn-loss function is to minimise pn-distance.

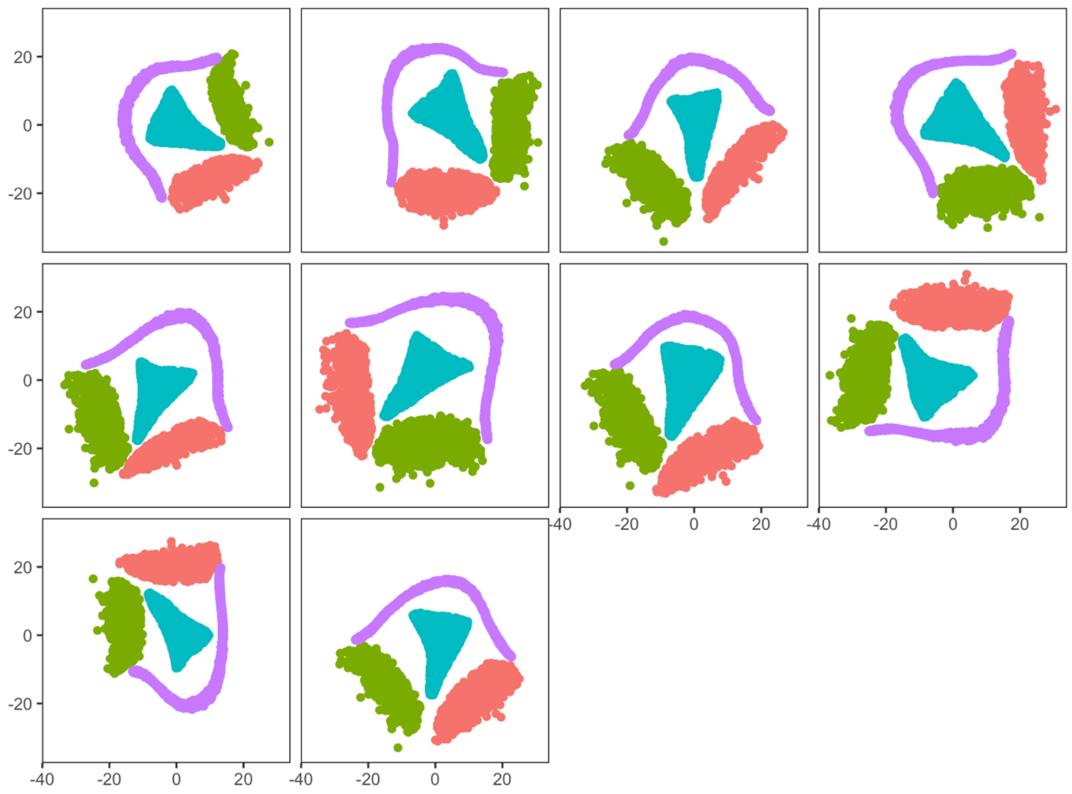

Supplementary Figure S2. ivis embeddings on the repeated ten runs on the simulated Smiley dataset (n = 5,000 points). Each point is colored according to its assigned cluster.

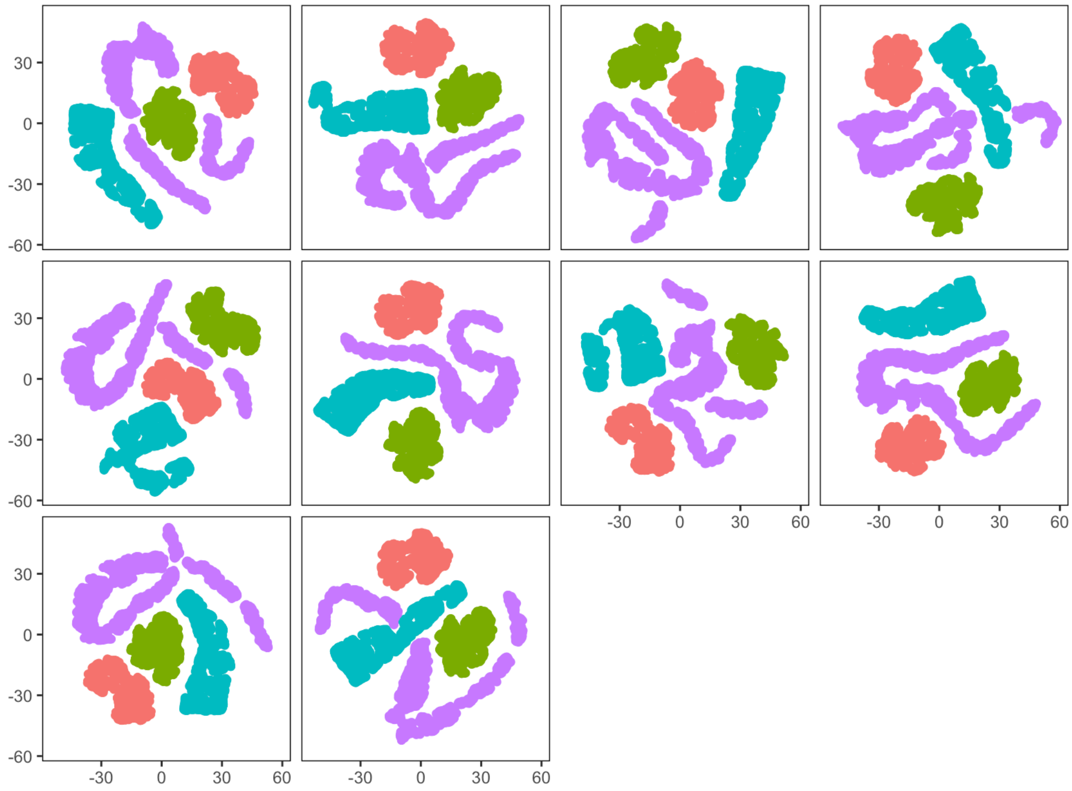

Supplementary Figure S3. t-SNE embedding on the repeated ten runs on the simulated Smiley dataset (n = 5,000 points). Each points is colored according to its assigned cluster.

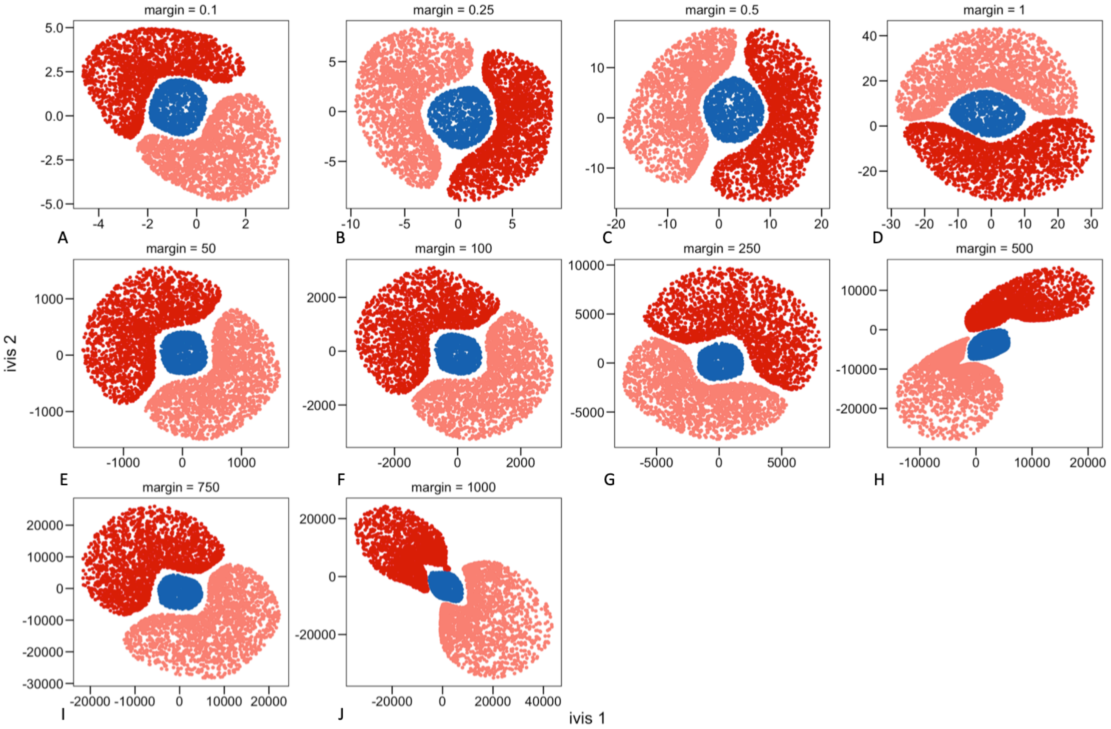

Supplementary Figure S4. Effects of the margin (*m*) hyperparameter on ivis embeddings of the Cassini dataset (n = 5,000 data points). Each point is colored according to its assigned cluster

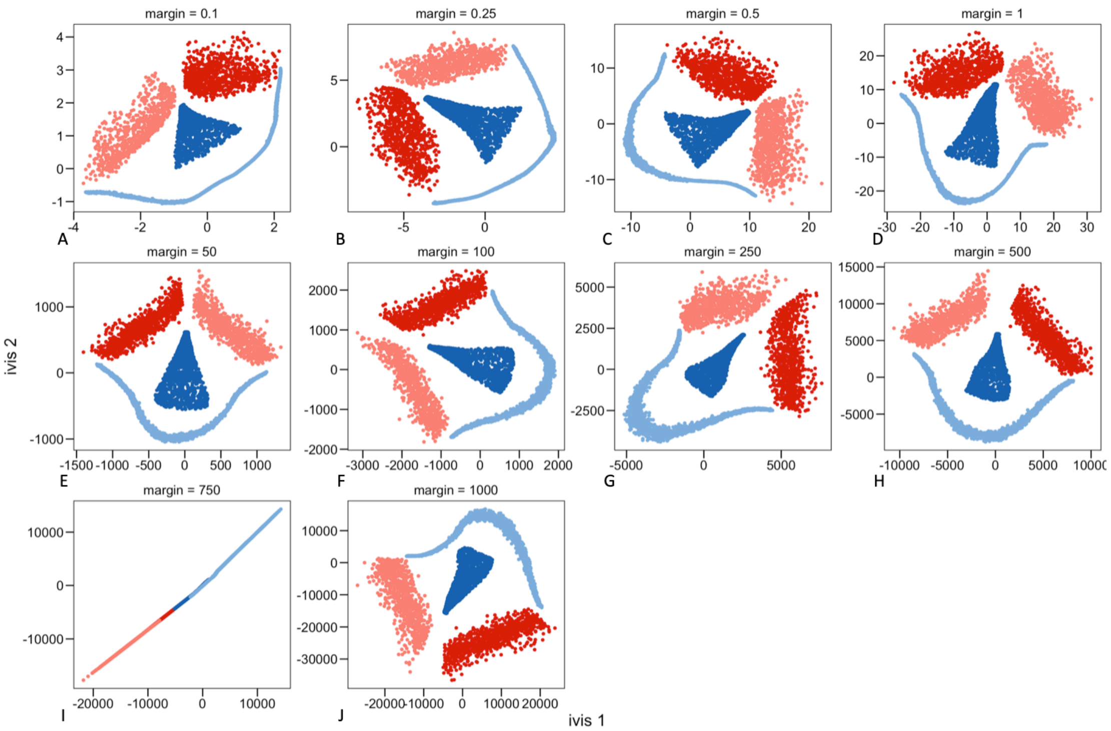

Supplementary Figure S5. Effects of the margin (*m*) hyperparameter on ivis embeddings of the Smiley dataset (n = 5,000 data points). Each point is colored according to its assigned cluster

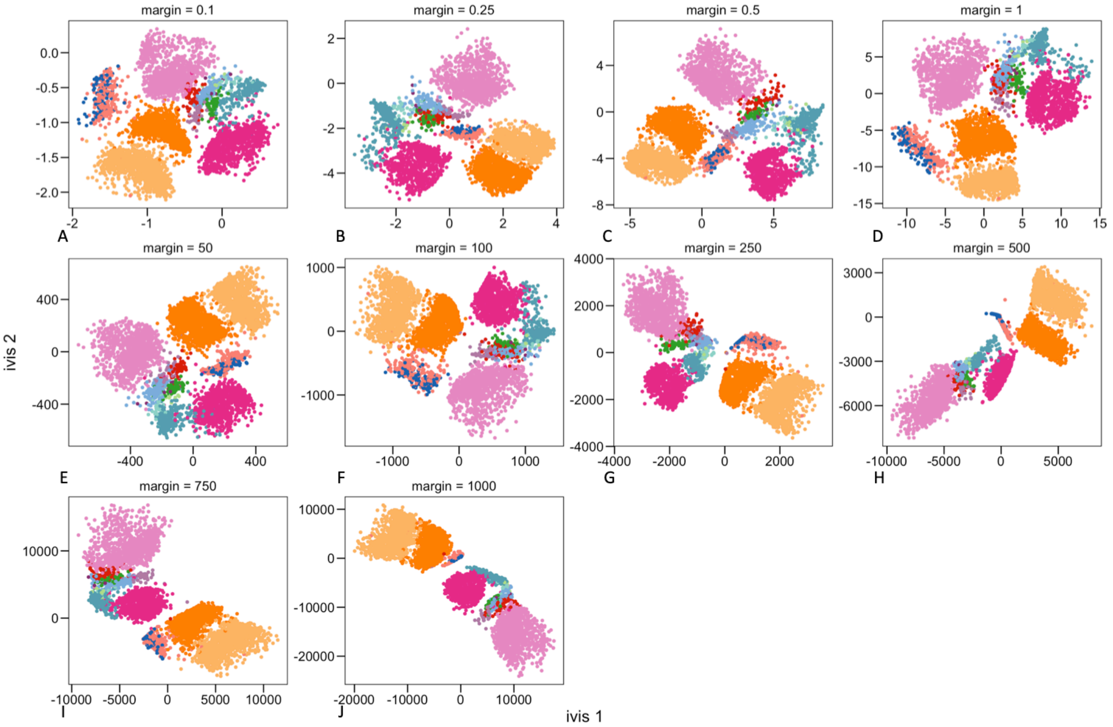

Supplementary Figure S6. Effects of the margin (*m*) hyperparameter on ivis embeddings of the BMMC dataset (n = 5,000 data points). Each point is colored according to its assigned cluster

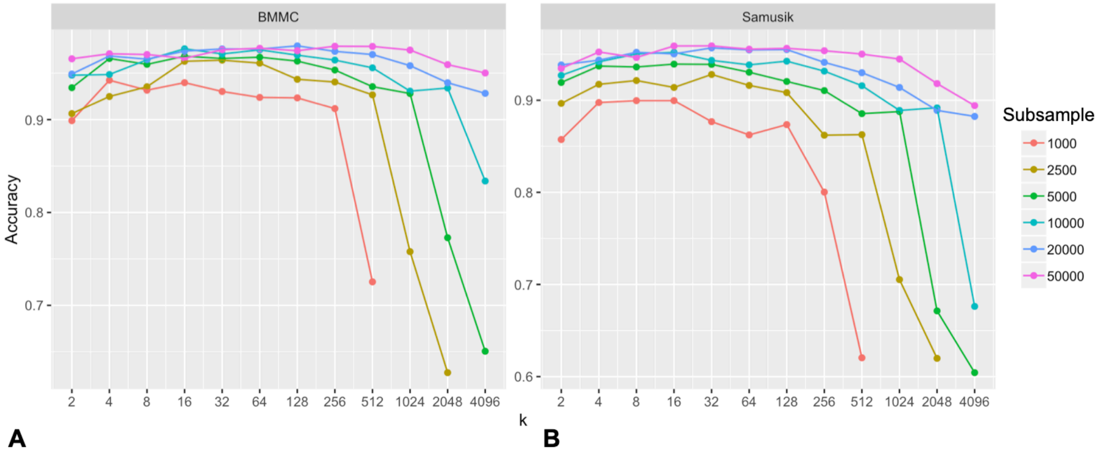

Supplementary Figure S7. Effects of *k* values on the accuracy of identification of manual gates using ivis embeddings of varying sizes (subsamples) in the BMMC (**A**) and Samusik (**B**) datasets.

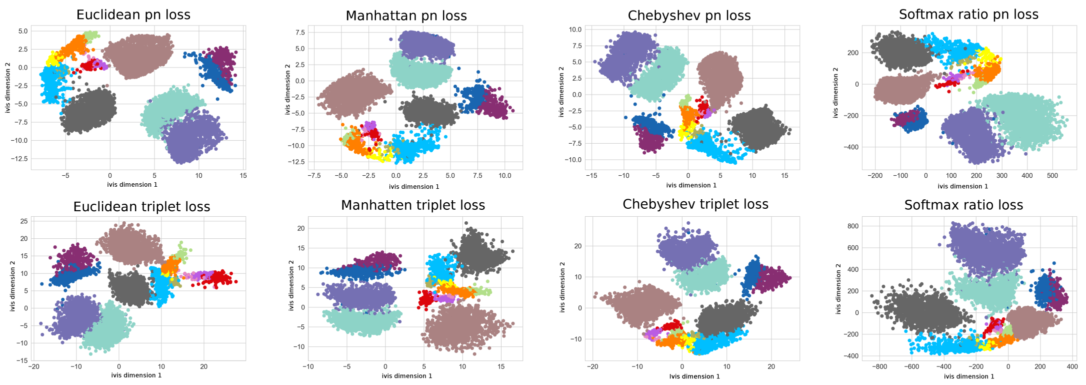

Figure S8. Comparison of two-dimensional ivis embeddings of a random subset of 10,000 rows from the BMMC dataset using various loss functions and distance metrics. Data-points colored according to manual gates. ivis hyperparameters were held constant across runs at their default values while the loss function and distance metric were varied. Standard triplet loss, pn loss, and softmax ratio loss were compared as well as Euclidean distance, Manhattan distance, and Chebyshev distance metrics.

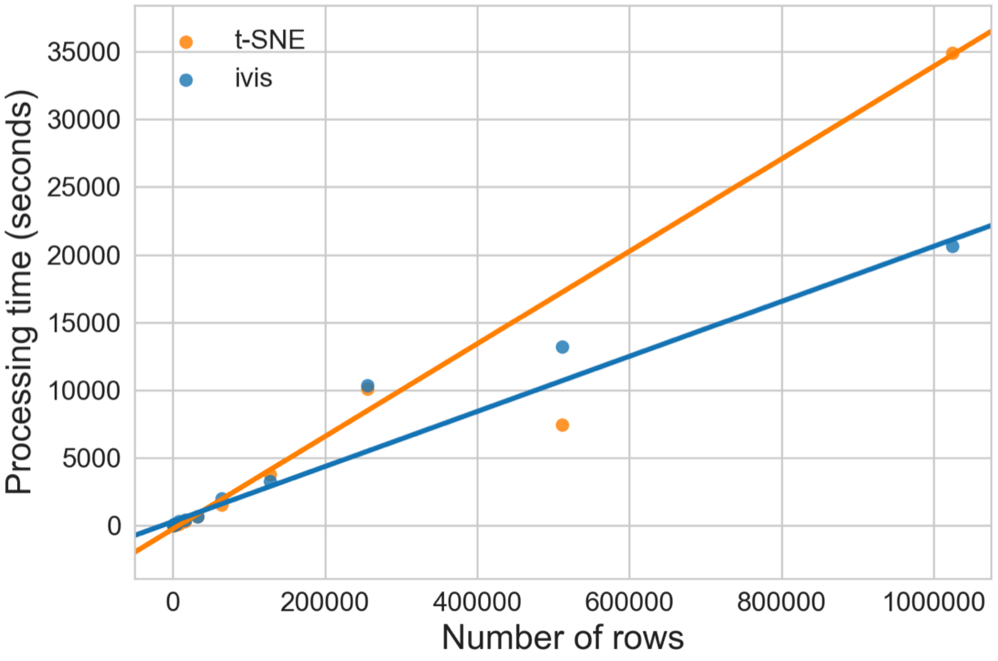

Supplementary Figure S9. Time complexity of ivis and t-SNE algorithms. sklearn library's implementation of Barnes-Hut t-SNE was used for all calculations. The input dataset consisted of 32 features, generated using isotropic Gaussian distribution.
